# Life-threatening check valve formation due to tracheobronchial aspergillosis

**DOI:** 10.1186/s40981-015-0022-5

**Published:** 2015-10-16

**Authors:** Hideki Matsuura, Satoki Inoue, Kazuaki Atagi, Masahiko Kawaguchi

**Affiliations:** Division of Intensive Care and Department of Anesthesiology, Nara Medical University, 840 Shijo-cho, Kashihara, Nara 634-8522 Japan

**Keywords:** Aspergillosis, Check valve, Air trapping

## Abstract

A 12-year-old girl receiving chemotherapy for acute myeloid leukemia had a fever of unknown origin in spite of administration of micafungin. Her respiratory condition suddenly deteriorated. Her trachea was intubated, and positive pressure ventilation was initiated; however, her respiratory condition further deteriorated. Expiratory volume was considerably lower than inspiratory volume. Simultaneously, she developed severe hypotension and bradycardia, and tension pneumothorax was suspected. Emergent chest decompression was subsequently performed; however, her airway resistance was still high. Bronchoscopy was performed to remove a foreign body in the carina. Subsequently, her respiratory status improved. Histopathological examination revealed that the foreign body was a fibrinous blood clot mixed with fungal hyphae of *Aspergillus niger*. Life-threatening check valve formation due to tracheobronchial aspergillosis under positive-pressure ventilation may be rare; however, once it occurs, prompt establishment of an escape route for trapped air, such as thoracentesis, may be required.

## Background

Tracheobronchial aspergillosis, an uncommon form of *Aspergillus*-related lung disease, is mainly observed in immunocompromised patients with acquired immunodeficiency disorder and those undergoing immunosuppressive therapy for hematological malignancies or solid organ transplantation [1, 2]. Large airway obstruction caused by invasive tracheobronchial aspergillosis may lead to respiratory failure. Here we present a case of a patient with a life-threatening check valve formation due to tracheobronchial aspergillosis under positive-pressure ventilation.

## Case presentation

**Fig. 1:**
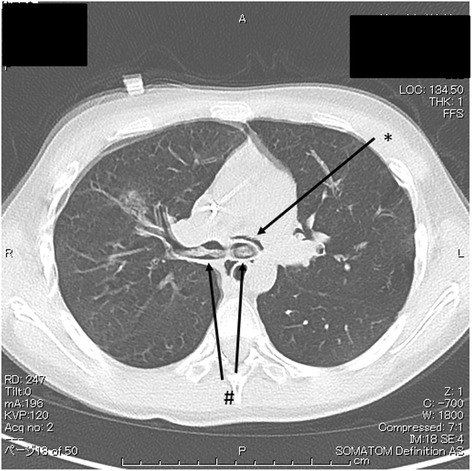
A chest CT before ICU admission. The arrows show a large high-density lesion in the carina over the bilateral bronchi (#) and mediastinal emphysema (*)

**Fig. 2:**
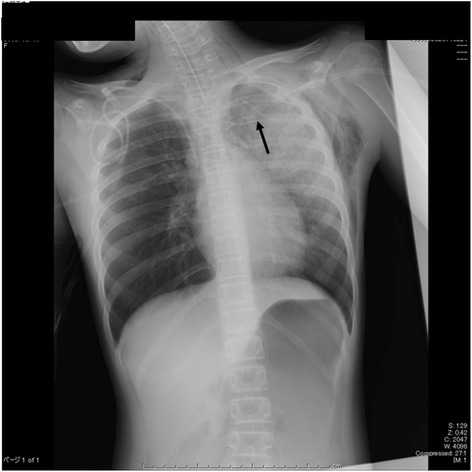
A chest X-ray after emergent chest decompression. The arrow shows the 18G intravenous cannula for chest decompression

**Fig. 3:**
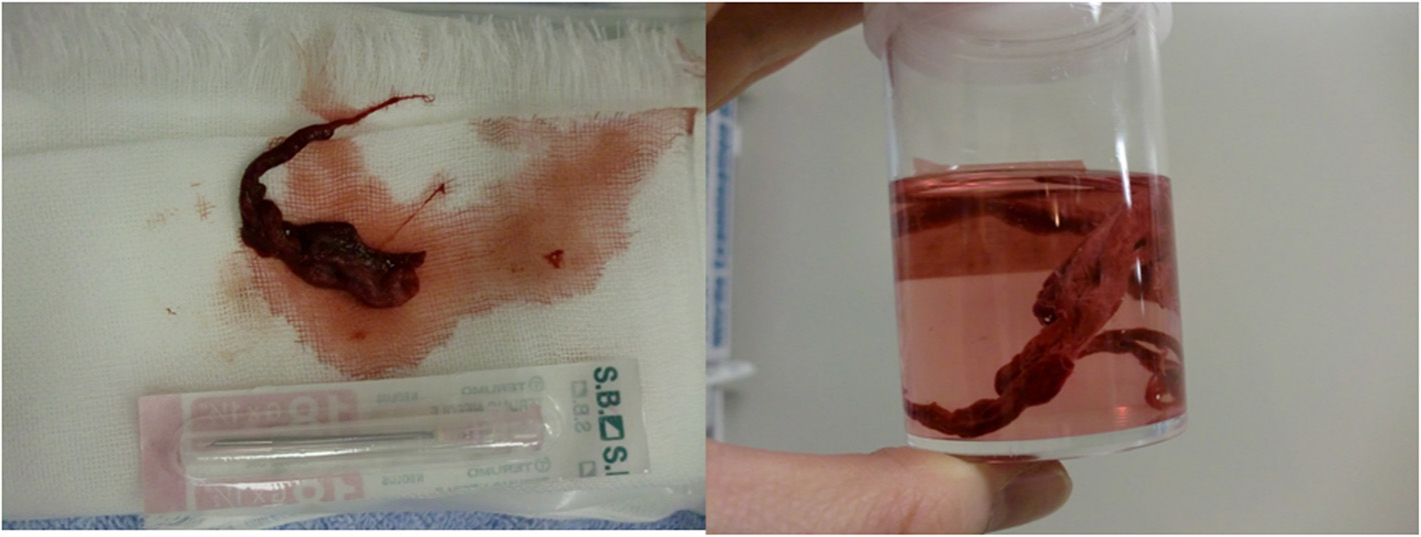
The foreign body removed by bronchoscopy

**Fig. 4:**
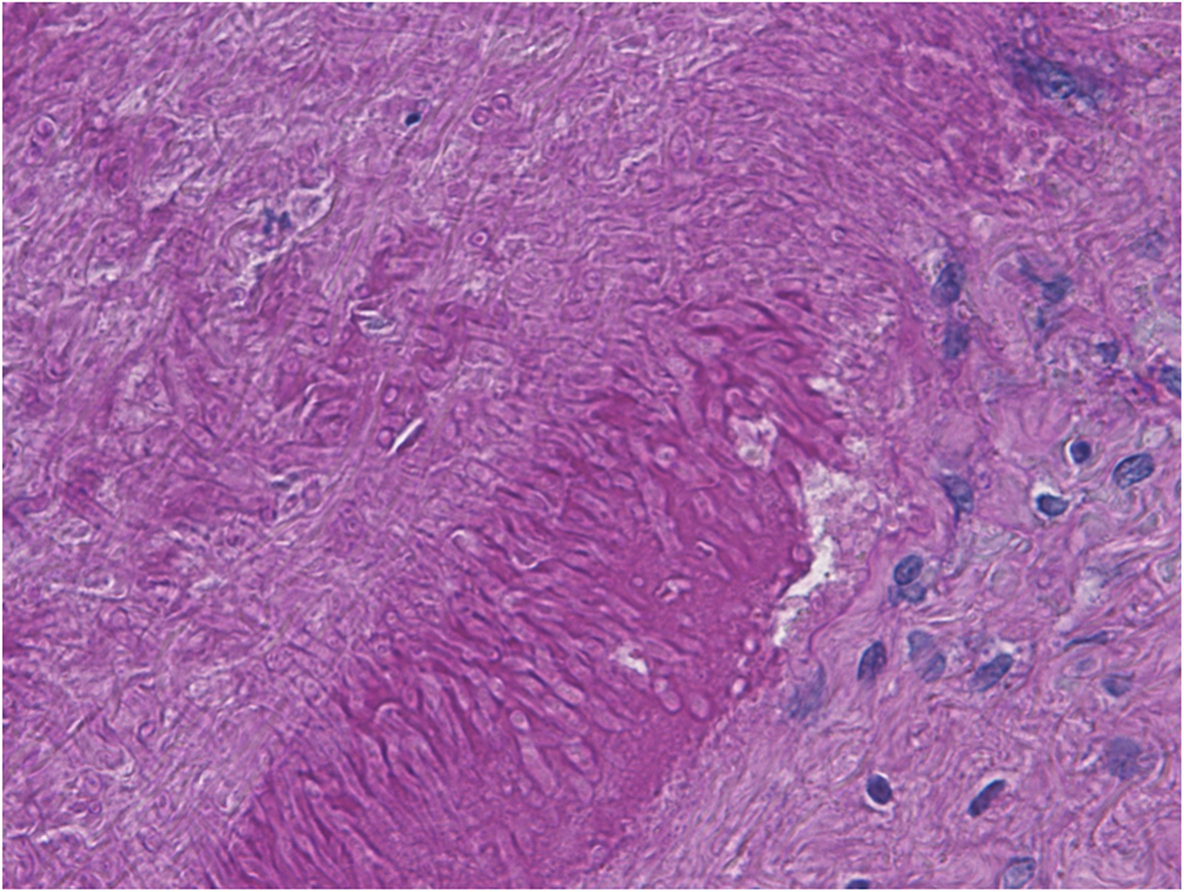
The histological examination for the foreign body. The histological examination shows numerous hyphae with background blood debris (hematoxylin and eosin staining)

We obtained approval from the institutional review board (Nara Medical University No.860) and written informed consent from the family for this case report. A 12-year-old girl receiving chemotherapy for acute myeloid leukemia had fever of unknown origin in spite of administration of broad-spectrum antibiotics. Micafungin administration was initiated because a fungal infection was suspected. Her regular chest X-ray revealed slight lung expansion but was otherwise almost normal. On the same day when X-ray examination was performed, she suddenly coughed up blood, and her respiratory condition deteriorated. An urgent chest CT was performed, which revealed a large high-density lesion in the carina over the bilateral bronchi and mediastinal emphysema (Fig. 1). Subsequently, she was admitted to the intensive care unit because of respiratory failure refractory to oxygen therapy. She became severely dyspneic with a respiratory rate of 40–50 breaths per minute. On admission, her SpO_2_ level with 15 L/min oxygen was approximately 80 %, which was improved using a bag valve mask ventilation. Therefore, it was assessed that she required immediate tracheal intubation and positive-pressure ventilation. After administering 5 mg midazolam, a cuffed 6.5-mm tracheal tube was inserted using a Macintosh-type laryngoscope, and manual positive-pressure ventilation was initiated. However, airway resistance was high, and expiratory volume was considerably lower than inspiratory volume, although an air leak around the tracheal tube was negligible. Meanwhile, her SpO_2_ level decreased and could not be detected, and her heart rate decreased from 140 bpm to 30 bpm. It was difficult to palpate her carotid. It appeared that her chest, particularly on the left side, expanded. Therefore, we determined that she developed left-sided tension pneumothorax. Subsequently, emergent chest decompression with an 18G intravenous cannula was performed at the second or third rib space in the mid-clavicular line. An immediate rush of air out of the chest was confirmed. Her SpO_2_ level became detectable, and the carotid was palpable. After confirming the release of the tension pneumothorax (Fig. 2), a regular chest drain tube was replaced with the intravenous cannula. However, her airway resistance was still high. During the course, neuromuscular block was not achieved so that her spontaneous breathing could be maintained; however, her breath was very weak. At that time, her blood gas analysis revealed pH = 7.000, PaCO_2_ = 88 Torr, PaO_2_ = 124 Torr, and HCO3^−^ = 21 mmol/L with the fraction of inspiratory oxygen = 70 % under manual bag ventilation. Repeated blind tracheal suctioning was ineffective, leading us to perform bronchoscopy to remove the foreign body in the carina. A foreign body-like blood clot in the carina dominantly to the left bronchus was observed using a flexible bronchoscopy. The foreign body was removed en bloc with the tracheal tube because it was too large to pass through the tracheal tube (Fig. 3). After successfully removing the foreign body, her respiratory status gradually improved, and she was finally weaned from mechanical ventilation under amphotericin B administration. Her infectious status was also under control with voriconazole medication. Afterward, histopathological examination revealed that the foreign body was a fibrinous blood clot mixed with numerous filamentous fungal hyphae of *Aspergillus niger* (Fig. 4).

## Discussion

Invasive pulmonary aspergillosis usually develops in severely immunocompromised patients, such as transplantation recipients, those with malignancy, or those with acquired immunodeficiency disorders. Among these patients, tracheobronchial aspergillosis is rare [1, 2]. It has been suggested that invasive tracheobronchial aspergillosis may lead to fatal hemorrhage because of the development of a bronchovascular fistula [1, 2]. In this case, the tracheobronchial blood clot may have been due to the development of a bronchovascular fistula. However, we cannot be certain of this fact.

The check valve mechanism of airway obstruction is a condition usually associated with the aspiration of an exogenous foreign body [3]. However, in this case, the cause of the tension pneumothorax was considered to be the check valve formation by a blood clot mixed with numerous fungal hyphae in the carina. Brennan et al. reported a case of check valve airway obstruction by a blood clot [3]. In addition, Kadota et al. suggested that the check valve mechanism caused by a presence of granulation tissue plugging the bronchiole lumens may be a leading cause of pneumothorax and cyst formation in patients with organizing pneumonia [4]. Moreover, Söllmann et al. reported a blood clot-like foreign body interspersed with *Aspergillus* hyphae obstructing the distal chip of the tracheal tube with a check valve-like formation, which led to tension pneumothorax after anesthesia induction [5]. In this case, it is reasonable to believe that the blood clot composed of numerous fungal hyphae in the carina caused the check valve mechanism of the airway, which led to overextension of the lung and eventually to tension pneumothorax. This leading mechanism for tension pneumothorax may have been accelerated by positive-pressure ventilation; however, tension pneumothorax itself could have already developed under spontaneous breathing prior to positive-pressure ventilation because of the strength of the patient’s respiratory effort.

In this case, it is clear that suffocation due to complete airway occlusion could be avoided by positive-pressure ventilation; however, failure in obtaining an escape route for the trapped air was a serious problem. Although the risk of positive pressure ventilation causing distal air trapping by a check valve mechanism has been suggested in case of a foreign body [6, 7], it has also been suggested that there is no clear clinical evidence in the literature surveyed to support this as a practical concern [8]. Therefore, no consensus has been reached regarding the development of critical air trapping due to a check valve formation. When expiratory volume is largely mismatched with inspiratory volume due to a check valve mechanism, severe air trapping can be a leading cause of cardiopulmonary failure, whether it is tension pneumothorax or lung hyperinflation. Moreover, it may be controversial to initiate emergent chest decompression prior to confirmation of tension pneumothorax. Recently, a case report suggested that needle thoracocentesis, particularly at the second rib space in the mid-clavicular line, had high failure rates and potentially serious complications [9]. However, it may be reasonable to consider thoracentesis, which has been recently recommended to be performed in the mid-anterior axillary line of the third–fifth intercostal space [9], when cardiopulmonary failure is accompanied by air trapping by a check valve mechanism. In severe cases of cardiopulmonary failure due to complete airway obstruction by a foreign body, extracorporeal membrane oxygenation may be considered according to expert opinions [10]. However, there is no guideline or expert opinion available for cardiopulmonary failure by air trapping due to a check valve mechanism. In such a case, it is worthwhile to attempt thoracentesis prior to cardiopulmonary bypass so that no time is lost in establishing an escape route for trapped air. This may be the case particularly in patients with strongly suspected airway bleeding, such as the circumstance in this case.

## Conclusions

Life-threatening check valve formation due to tracheobronchial aspergillosis under positive-pressure ventilation may be rare. However, once it occurs, prompt establishment of an escape route for trapped air, such as thoracentesis, may be required.

## Consent

Written informed consent was obtained from the family for publication of this case report and any accompanying images. A copy of the written consent is available for review by the Editor-in-Chief of this journal.

## Abbreviations

SpO_2_: Saturation of peripheral oxygen
CT: Computed tomography
ICU: Intensive Care Unit
